# Cerebellar contributions to sequence prediction in verbal working memory

**DOI:** 10.1007/s00429-018-1784-0

**Published:** 2018-11-02

**Authors:** Jutta Peterburs, Laura C. Blevins, Yi-Shin Sheu, John E. Desmond

**Affiliations:** 1 Department of Neurology, Division of Cognitive Neuroscience, Johns Hopkins University School of Medicine, Baltimore, MD, USA; 2 Department of Biological Psychology, Institute of Experimental Psychology, Heinrich-Heine-University, Düsseldorf, Germany; 3 Department of Psychology, American University, Washington, DC, USA

**Keywords:** Cerebellum, Verbal working memory, Cognition, fMRI, Prediction, Sequence learning, Sequence detection

## Abstract

Verbal working memory is one of the most studied non-motor functions with robust cerebellar involvement. While the superior cerebellum (lobule VI) has been associated with articulatory control, the inferior cerebellum (lobule VIIIa) has been linked to phonological storage. The present study was aimed to elucidate the differential roles of these regions by investigating whether the cerebellum might contribute to verbal working memory via predictions based on sequence learning/detection. 19 healthy adult subjects completed an fMRI-based Sternberg task which included repeating and novel letter sequences that were phonologically similar or dissimilar. It was hypothesized that learning a repeating sequence of study letters would reduce phonological storage demand and associated right inferior cerebellar activations and that this effect would be modulated by phonological similarity of the study letters. Specifically, while increased phonological storage demand due to high phonological similarity was expected to be reflected in increased right inferior cerebellar activations for similar relative to dissimilar study letters, the reduction in activation for repeating relative to novel sequences was expected to be more profound for phonologically similar than for dissimilar study letters, especially at higher memory load. Results confirmed the typical effects of cognitive load (5 vs. 2 study letters) and phonological similarity in several cerebellar and neocortical brain regions as well as in behavioral data (accuracy and response time). Importantly, activations in superior and inferior cerebellar regions were differentially modulated as a function of similarity and sequence novelty, indicating that particularly lobule VIIIa may contribute to verbal working memory by generating predictions of letter sequences that reduce the likelihood of phonological loop failure before stored items need to be retrieved. The present study is consistent with other investigations that support prediction, which can be based on sequence learning or detection, as an overarching cerebellar function.

## Introduction

Recent research has highlighted a role of the cerebellum not only in motor behavior but also in cognition. Indeed, a growing number of neuroimaging and patient studies have provided evidence for cerebellar involvement in domains such as verbal working memory (e.g., Desmond et al. 1997; Justus et al. 2005; Chen and Desmond 2005a; Ravizza et al. 2006; Hayter et al. 2007; Chein and Fiez 2010; Peterburs et al. 2010, 2016; Marvel and Desmond 2010a; Stoodley 2012), associative learning (e.g., Topka et al. 1993; Ramnani et al. 2000; Toni et al. 2001; Gerwig et al. 2003; Cheng et al. 2008; Thoma et al. 2008; Timmann et al. 2010), executive function (e.g., Grafman et al. 1992; Rao et al. 1997; Karatekin et al. 2000; Bellebaum and Daum 2007; Richter et al. 2007; Balsters et al. 2013), language (e.g., Ackermann et al. 2007; Marien et al. 2014), emotional processing and emotion regulation (e.g., Schmahmann et al. 2007; for an overview, see recent consensus paper; Adamaszek et al. 2017), and error and feedback processing (e.g., Rustemeier et al. 2016; Von der Gablentz et al. 2015; Peterburs et al. 2012, 2015). However, there is currently no consensus about how the cerebellum contributes to these functions. It has been proposed that, due to its uniform neuro-architecture with closed input–output loops that connect cerebellum and cerebrum (Middleton and Strick 1994; Strick et al. 2009), the cerebellum also possesses uniform processing habits to enable overarching, domain-independent functions such as monitoring, coordination, and timing (Strick et al. 2009). Along these lines, the cerebellum may also provide other basic functions such as sensory acquisition (Gao et al. 1996; Bower 1997; Shih et al. 2009), internal modelling/error correction (Wolpert et al. 1998; Ito 2008), performance monitoring (Peterburs and Desmond 2016), and sequence detection (Braitenberg et al. 1997; Molinari et al. 2008; Leggio and Molinari 2015; Tedesco et al. 2017).

One of the most-studied non-motor functions with robust cerebellar involvement is working memory, and one of the experimental paradigms commonly used to study verbal working memory is a variant of the Sternberg Task (Sternberg 1966) in which subjects are presented with strings of study letters in the initial encoding phase, which they rehearse for several seconds (maintenance phase) while waiting for the presentation of a probe letter, which they then have to match to the initially presented study letters (retrieval phase). Previous investigations have revealed two cerebellar regions that exhibit activation during this task, a superior region localized in lobule VI and crus I, and an inferior region found in lobules VIII and VIIB (Desmond et al. 1997; Chein and Fiez 2001; Chen and Desmond 2005a, b; Durisko and Fiez 2010). The superior cerebellar region, along with posterior frontal regions, exhibited peak activation during the encoding phase of the task (Chen and Desmond 2005b; Chein and Fiez 2001). Activation in the superior cerebellum was also observed during a control task that was designed to mimic the motoric aspects of articulatory rehearsal but did not require any storage of verbal information (Chen and Desmond 2005a), thus suggesting that activation in lobule VI reflected articulatory control. In contrast, the inferior cerebellar region exhibited activation that peaked in the maintenance phase of the task but did not show activation during the motoric control task, and thus, appeared to be related to the phonological storage requirements of the task rather than articulation per se (Desmond et al. 1997; Chen and Desmond 2005a). Subsequent neuropsychological studies have demonstrated that cerebellar damage produces abnormalities in phonological storage-related phenomena such as the phonological similarity effect (Justus et al. 2005; Kirschen et al. 2008), and studies with cerebellar patients have specifically associated the inferior cerebellum with such abnormalities (Kirschen et al. 2008; Chiricozzi et al. 2008).

If the inferior cerebellum is in fact involved in phonological storage requirements of verbal working memory, then activation in this region should be sensitive to any increases in phonological storage demand or difficulty. In the present investigation, we manipulate phonological storage demand by presenting to subjects either phonologically similar or dissimilar letters, and we hypothesize that inferior cerebellar activation will be greater during the more demanding phonologically similar condition.

However, regardless of phonological demand of the stimuli, the question remains as to how the cerebellum actually contributes to the verbal working memory task. Forward model theories of cerebellar function (Wolpert et al. 1998; Ito 2008) posit that commands from neocortical regions— which can be either motor or cognitive in nature, and in the case of working memory would be a command to rehearse the letter sequence—are sent to the cerebellum in an efference copy of the command via the massive cortico-pontocerebellar projections. The cerebellum is hypothesized to develop a rapid prediction of the desired motor or cognitive trajectory, along with the sensory consequences of that trajectory. If the predicted sensory consequences fail to match the actual sensory consequences, climbing fiber signals are delivered to alter synaptic plasticity at the Purkinje cells to improve subsequent predictions (Wolpert et al. 1998; Ito 2008). In verbal working memory, two predictions would be useful to neocortical regions involved in the task, namely predictions of the articulatory trajectory for rehearsing the letter sequence, and predictions of the phonological stream derived from the rehearsal process. Such predictions could decrease the likelihood of phonological loop failure prior to the utilization of the information during the retrieval phase of the task.

Thus, from a forward model architecture, the cerebellum might contribute to verbal working memory by generating predictions of the sequence of letters that need to be rehearsed, a view that is consistent with those of other investigators who have emphasized that sequence detection may be a primary function of cerebellar physiology (Leggio et al. 2011; Molinari et al. 2008).

Previous work has provided evidence for cerebellar sequence detection in the somatosensory domain, with absent or abnormal somatosensory mismatch negativity in patients with cerebellar lesions (Restuccia et al. 2007), or in the language domain in terms of sequencing of syllable strings (Ackermann et al. 2007). Interestingly, cerebellar dysfunction, reflected for instance in impaired reproduction and learning of (motor) sequences as well as reduced cerebellar activations associated with these functions (Nicolson et al. 1999), has been proposed to underlie dyslexia (e.g., Fawcett et al. 1996; see Nicolson and Fawcett 2011, for a review). More recently, the cerebellum, along with auditory, inferior frontal, and parietal areas, has been implicated in the prediction of own and partner musical sequences after short-term piano duet training (Lappe et al. 2017). Furthermore, patients with cerebellar lesions were shown to exhibit impaired cognitive sequencing of verbal or pictorial material, depending on lesion laterality (Leggio et al. 2008).

To examine the possibility that the cerebellum contributes to verbal working memory via sequence prediction, the present study applied a Sternberg verbal working memory task that included repeating and novel sequences of study letters for both phonologically similar and dissimilar study letters. With regard to behavior, it was hypothesized that phonological similarity would be associated with decreased accuracy and increased response times (RTs; Baddeley 1965; Conrad 1964; Sweet et al. 2008). Moreover, based on previous work showing learning of perceptual sequences in the absence of motor sequences (e.g., Dennis et al. 2006), RTs were expected to decrease over the course of the task for trials with repeating sequences of letters but not for trials with all novel sequences, reflecting implicit acquisition of the repeating sequences. With regard to neural responses, learning a repeating sequence of study letters should reduce phonological storage demand and associated right inferior cerebellar activations relative to novel study letter sequences. Furthermore, it was hypothesized that this effect would be modulated by phonological similarity of the study letters. Specifically, while increased phonological storage demand due to high phonological similarity was expected to be reflected in increased right inferior cerebellar activations for similar relative to dissimilar study letters, the reduction in activation for repeating relative to novel sequences was expected to be more profound for phonologically similar than for dissimilar study letters, especially at higher memory load.

## Methods

### Subjects

Twenty healthy adult volunteers (13 female, 7 male; mean age 25.1 ± 2.9 years, age range 19–30 years) were recruited from the Baltimore community. All subjects were native English speakers, right-handed according to self-report, and had normal or corrected-to-normal vision. Exclusion criteria were current or past neurological or psychiatric illnesses or head trauma, current medication affecting the central nervous system, and further criteria pertaining to MRI scanning, i.e., (self-reported) claustrophobia, implanted electric or ferromagnetic devices, and pregnancy. Mean educational attainment was 17.2 ± 1.6 years (range 14–20). All subjects gave written informed consent prior to participation and received monetary compensation for participation and travel expenses. The study conforms to the Declaration of Helsinki and was approved by the Johns Hopkins School of Medicine Institutional Review Board.

### Sternberg verbal working memory task

**Fig. 1:**
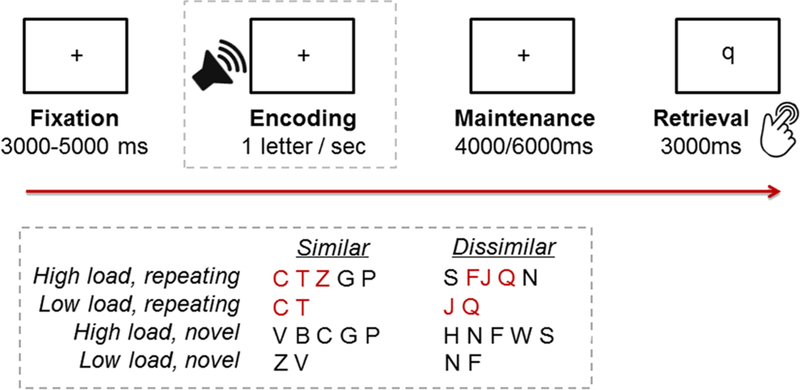
Time course of stimulus presentation and examples of study letters according to phonological similarity (similar/dissimilar), load (low/high), and novelty (repeating sequence/novel)

In the task variant used in the present study, encoding stimuli were digitally recorded spoken letters pronounced by a male actor that was downloaded from a royalty-free website (soundbible.com/2009-A-Z-Vocalized.html). In accordance with the procedure applied in a previous study (Kirschen et al. 2010), on each trial, either two (low load) or five (high load) of these letters (all consonants) were presented binaurally at one item per s. Probe letters were visually presented lower case letters presented for 3 s. To manipulate rehearsal demand during the maintenance phase, letters were either drawn from a pool of phonologically similar (B–C–D–G–P–T–V–Z) or a pool of phonologically dissimilar (F–H–J–N–Q–R–S–W) items. Moreover, half of the trials contained a repeating sequence of three letters (C–T–Z for similar and F–J–Q for dissimilar). For the high-load condition, the repeating sequence could appear at the beginning, middle, or end of the five-letter array. For low-load trials, i.e., trials with two study letters, only parts of the sequences (C–T or T–Z, and F–J or J–Q) were used. Figure 1 illustrates the time course of stimulus presentation in the task. At the beginning of each trial, a fixation cross was presented for 3–5 s. In the ensuing encoding phase, two or five study letters were presented sequentially via noise canceling MR-compatible headphones (OptoActive II™, Optoacoustics Ltd., Moshav Mazor, Israel). Noise cancelation headphones allowed scanner noises to be reduced to 70–77 dB; sound output during stimulus presentation was calibrated to 85 dB. The maintenance phase during which the study letters were rehearsed while a blank screen was presented lasted 4–6 s. In the subsequent retrieval phase, the probe was presented for 3 s, and subjects indicated “match” or “non-match” by pressing one of two response buttons with their right index or middle finger. Subjects were instructed to respond as fast and as accurately as possible. Response time (RT) and accuracy were recorded for each trial. To ensure that subjects were familiar with the task, four practice trials containing study letters that were not part of the similar or dissimilar letter pools used for the actual experiment were completed outside the scanner prior to starting the experiment.

The task comprised 3 runs of 72 trials, amounting to a total of 216 trials. Phonological similarity (similar or dissimilar), novelty (repeating sequence or novel), and cognitive load (high or low) were counterbalanced within each run. Furthermore, trial order was pseudorandomized so that presentation of identical parameters was limited to three consecutive trials and so that the probe on any given trial had not been part of the study letters in the previous trial. The sequential position of the repeating sequence was also balanced across trial types within each of the task runs. Each run contained 32 match and 32 no-match trials as well as eight trials without a probe. No-probe trials were included to allow the hemodynamic response to fully return to baseline following the maintenance delay. In no-probe trials, subjects viewed a blank screen throughout the retrieval phase, and no response was expected. The probe letter was a member of the repeating sequence 50% of the time for each trial type in each run.

Stimulus presentation was controlled with E-Prime 2 software (Psychology Software Tools Inc., Sharpsburg, PA, USA). Stimuli were presented on a Hewlett Packard xw4300 workstation running Windows 7. The visual display was rear-projected onto a screen in the MRI scanner located behind the subject’s head and reflected onto a mirror within the subject’s line of view that was fixed to the head coil. Responses were collected using two fiber optic button boxes (MRA, Inc., Washington, PA).

### Analysis of behavioral data

Accuracy and median RTs on correct trials were analyzed by means of a 3 × 2 × 2 × 2 repeated-measures analyses of variance (ANOVAs) with run (1–3), load (high or low), novelty (sequence or novel), and similarity (similar or dissimilar) as within-subject factors. Greenhouse–Geisser correction was applied to account for sphericity violations when appropriate. Post hoc *t* tests were performed to resolve interactions. Effects of run were resolved by linear trend analysis. The significance level was set to *p* < 0.05.

### MRI data acquisition

MRI data were acquired using a 3.0T Philips Intera scanner (Philips, Eindhoven, NL). The structural MRI protocol consisted of a T1-weighted MPRAGE (TR = 7.0 ms; TE = 3.3 ms; TI = 982 ms; flip = 8°, voxel size = 0.83 mm × 0.83 mm; slice thickness = 1 mm; 170 sagittal slices; FOV = 240 mm × 240 mm; 1 NEX). FMRI data were collected using a T2-weighted gradient echo EPI pulse sequence (TR = 2000 ms; TE = 30 ms; flip = 76°; voxel size = 2.5 mm x 2.5 mm; slice thickness = 3 mm; gap = 2 mm; 35 ascending slices; FOV = 220.39 mm × 200.35 mm; 1 NEX). T2-weighted images were acquired in the obliqueaxial plane rotated 25° clockwise with respect to the AC–PC line to optimize imaging of the cerebellum and neocortex. 554 volumes were acquired per task run. The start of the fMRI scan was synchronized with the start of the experiment using E-prime software (Psychology Software Tools Inc., Sharpsburg, PA, USA) at the beginning of each run.

### Analysis of functional MRI data

The SPM12 software package (Wellcome Department of Cognitive Neurology, London, UK) was used for preprocessing and statistical computations. Standard image preprocessing steps were performed, including slice timing correction (reference = middle slice), motion correction, anatomical coregistration, normalization to the Montreal Neurological Institute (MNI) stereotaxic space, and spatial smoothing (FWHM = 5 mm). Furthermore, motion-related artifacts and global mean outliers were identified with the Artifact Detection Tools (ART; https://www.nitrc.org/projects/artifact_detect/) software package and used as covariates of no interest. Individual statistical maps were computed for each subject using the general linear model approach as implemented in SPM12, with high-pass filtering of 128 s. Load, similarity and novelty were entered as factors. Although all encoding, maintenance, and retrieval events were modelled in the GLM analysis, because the present study was aimed to specifically elucidate cerebellar contributions to rehearsal processes in verbal working memory, MR analysis was limited to the maintenance phase of the Sternberg task. Random effects analyses were performed to map the average brain responses on correct trials only. Incorrect trials were not explicitly modelled and considered as residual variance. The GLM was estimated for each subject separately, and the resulting contrasts were entered into group-level random effects analysis using one-sample *t* tests against a contrast value of zero at every voxel at whole-brain level. The analysis strategy was to first identify all brain region clusters that exhibited a significant working memory load effect. To identify these clusters, we used a voxel-wise significance level of *p* < .001 and an FDR-corrected cluster significance of *p* < .05. Further analyses of phonological similarity and novelty were conducted only on an a priori set of right cerebellar, left frontal, and left parietal regions of interest (ROIs) exhibiting positive load effects (i.e., high load-activation > low-load activation), or left superior temporal ROIs exhibiting any load effects; the latter regions have been implicated in verbal working memory from either neuroimaging or patient investigations (e.g., Leff et al. 2009; Kirschen et al. 2010). This set comprised one superior temporal, two cerebellar, and four frontal regions. Repeated-measures ANOVAs on this set focused only on high-load stimuli to examine main effects and interaction of phonological similarity and novelty, and included four planned (a priori) comparisons: (1) SNH–SRH; (2) DNH–DRH; (3) SNH–DNH; and (4) SRH–DRH, where S means phonologically similar, D means phonologically dissimilar, N means novel sequence, R means repeating sequence, and H means high load (five letters). As described in “Introduction”, we hypothesized that comparisons (1) and (3) would be significant for the inferior cerebellum, indicating that this region is responsive to both phonological demand and sequence effects for phonologically demanding stimuli. In addition, because prior work discussed above suggest that superior and inferior cerebellar regions have different contributions to the verbal working memory task, we conducted a repeated-measures ANOVA with cerebellar region (inferior vs. superior), as well as phonological similarity and novelty as factors. We hypothesize that the different contributions of inferior and superior cerebellar regions would be evident in this analysis as a significant region × similarity × novelty interaction. For 14 remaining load-sensitive ROIs that were not included in the a priori set, subsequent analyses of phonological similarity and novelty used Bonferroni-corrected *p* values according to the number of regions analyzed, yielding a corrected *p* value of 0.0036.

For load-sensitive ROIs that were large, or spanned multiple anatomical regions, more focused regions of interest were created by restricting the ROI to a sphere of 10 mm radius that was centered on a local maximum for the cluster. In Table 1, the cluster with the peak in left postcentral gyrus (indicated with an “a” symbol in the table) had ROIs created from the local maxima in inferior frontal and precentral gyri. Similarly, the peak centered on left middle frontal gyrus (indicated with “b” in the table) had ROIs created from local maxima in medial and superior frontal gyri. The large activations in the left and right superior temporal gyri were also focused at their peak coordinates using a 10-mm radius sphere.

MNI coordinates were transformed into the coordinate system of the Talairach and Tourneaux stereotaxic atlas (Talairach and Tournoux 1988) using the MNI to Talairach transformation described by Lancaster et al. (1997) to make anatomical determinations of the neocortical activations. However, MNI coordinates are reported in the tables and figures. For the cerebellum, MNI coordinates were referenced with the SUIT atlas (Diedrichsen et al. 2009) and with a supplemental probabilistic atlas of human cerebellar nuclei (Dimitrova et al. 2006).

## Results

### Behavioral data: accuracy and reaction time

Although performance was generally high, data from one subject were excluded due to low performance (< 60% accuracy averaged across all three runs) in the high-loadsimilar-novel condition. All analyses thus included data from the remaining 19 individuals who performed above chance level (> 60% accuracy averaged across runs) in all conditions. None of the subjects reported having been aware of any repeating sequences of letters during debriefing after the experiment.

**Fig. 2:**
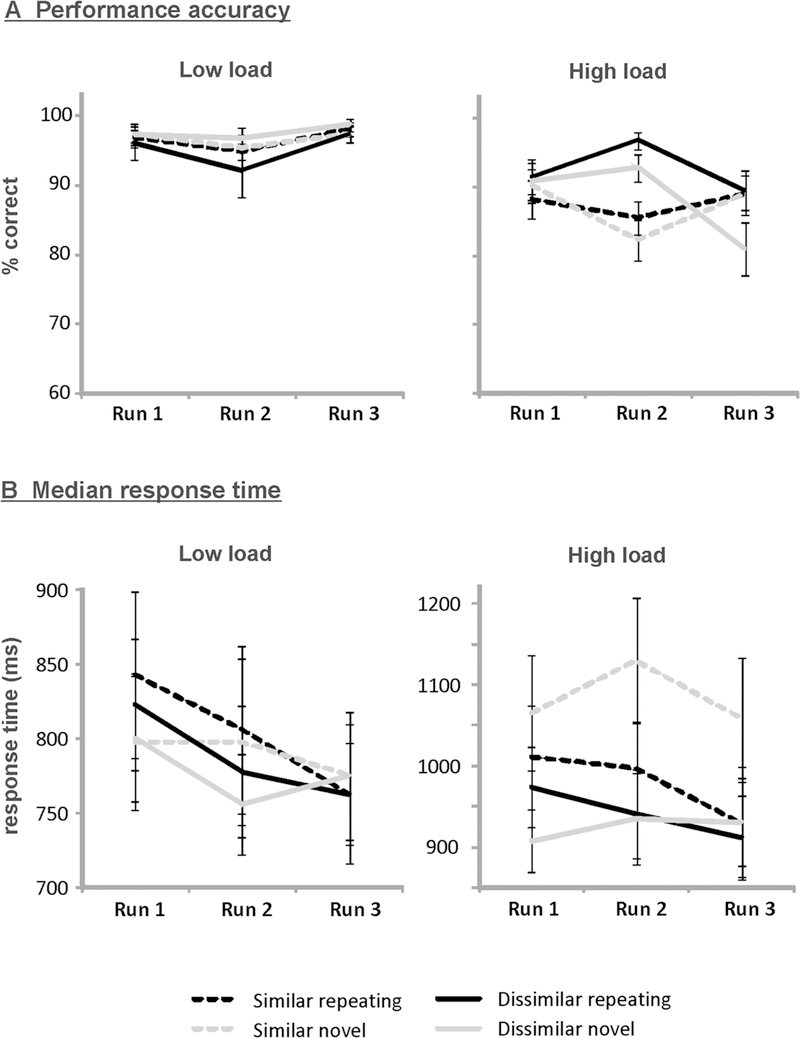
Mean performance accuracy (**a**) and median response time (**b**) according to run (1–3), similarity (similar/dissimilar), load (low/high), and novelty (repeating/novel)

Figure 2a provides mean performance accuracy according to run, similarity, load, and novelty. The ANOVA yielded significant main effects of similarity (*F*_[1,18]_ = 8.46, *p* = .009) and load (*F*_[1,18]_ = 48.30, *p* < .001), indicating that accuracy was higher for phonologically dissimilar as compared to similar trials, and for low- as compared to high-load trials. These effects were further qualified by a significant similarity by load interaction (*F*_[1,18]_ = 7.59, *p* = .013). Post hoc paired-sample *t* tests comparing performance for similar and dissimilar trials according to load yielded significantly higher accuracy for dissimilar than for similar highload trials (*t*_18_ = 3.20, *p* = .005). Accuracy did not differ between the similar and dissimilar condition for low-load trials (*p* = .807). The interactions between novelty and load (*F*_[1,18]_ = 4.29, *p* = .053), run and similarity (*F*_[2,33]_ = 2.81, *p* = .079), novelty and similarity (*F*_[1,18]_ = 3.53, *p* = .077), and run and load (*F*_[2,34]_ = 3.11, *p* = .060) merely approached significance, as did the run by similarity by load three-way interaction (*F*_[2,36]_ = 3.22, *p* = .058). All other effects did not reach significance (all *p* > .171).

Median RTs on correct trials according to run, similarity, load, and novelty are provided in Fig. 2b. For median RT, significant main effects of similarity (*F*_[1,18]_ = 10.90, *p* = .004) and load (*F*_[1,18]_ = 69.23, *p* < .001) emerged, indicating that RTs were shorter for phonologically dissimilar as compared to similar trials, and for low- as compared to high-load trials. In addition, there was a significant similarity by load interaction (*F*_[1,18]_ = 9.56, *p* = .006). Post hoc pairedsample *t* tests showed that RTs were shorter for dissimilar high-load compared to similar high-load trials (*t*_18_ = 3.66, *p* = .002), while there was no difference between similar and dissimilar for low-load trials (*p* = .122). Moreover, a significant run by novelty interaction emerged (*F*_[2,34]_ = 7.93, *p* = .002). Linear trend analysis revealed a significant linear decrease in median RT across the three runs for trials with repeating sequences (*F*_[1,18]_ = 5.82, *p* = .027) but not for trials with novel sequences (*p* = .715), thus reflecting implicit learning of the repeating sequence. Furthermore, the similarity by novelty interaction was significant (*F*_[1,18]_ = 10.32, *p* = .005), as were the novelty by load (*F*_[1,18]_ = 4.73, *p* = .043) and the similarity by novelty by load interaction (*F*_[1,18]_ = 7.43, *p* = .014). To resolve the three-way interaction, post hoc paired-sample *t* tests were performed, comparing median RTs on trials with repeating and novel sequences according to similarity and load. For similar high-load trials, the repeating sequence significantly decreased RTs by 17.9% relative to novel sequences (*t*_18_ = − 2.51, *p* = .022). A small but significant opposite pattern was found for dissimilar high-load trials, which showed a 4.1% increase in RT for repeating sequences (*t*_18_ = 2.12, *p* = .048), due mainly to unusually low RTs for dissimilar novel trials during run 1. For both similar and dissimilar low-load trials, RTs did not differ between the two novelty conditions (both *p* > .299).

### Imaging data

#### Whole brain analysis: main effect of load (high> low)

**Fig. 3:**
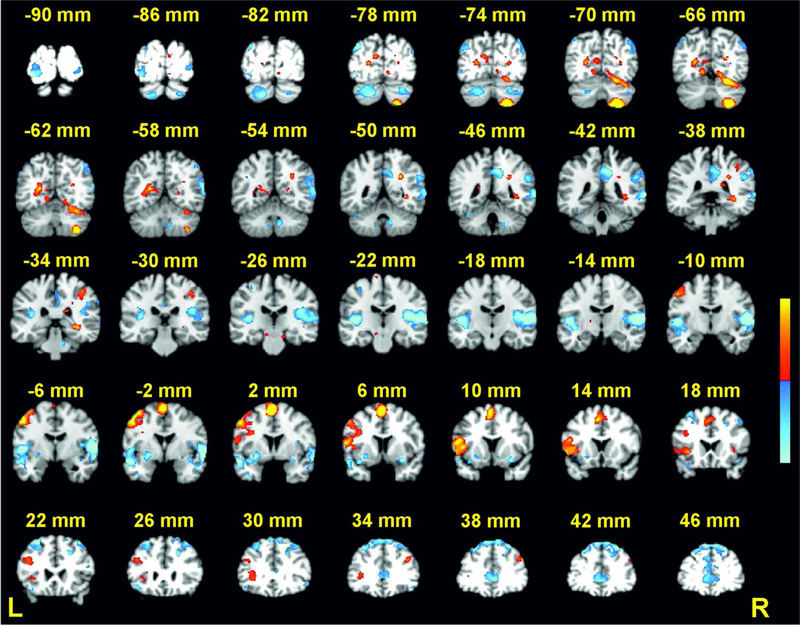
Activations for high vs. low cognitive load (peak coordinates provided in Table 1). Coronal slices from Talairach *y* =+46 to − 90 mm are depicted. Positive activations (high > low) are shown in red; negative activations (low > high) are shown in blue; *p* < .001 − .00001

BOLD signal changes for the load effect were observed in several cerebellar and neocortical regions (see Table 1; Fig. 3). Signal increase for high relative to low load (i.e., positive load effect) was found in right superior cerebellum (lobule VI), right inferior cerebellum (lobule VIIIa), right inferior parietal lobule (IPL), left inferior frontal and left medial frontal regions, left postcentral gyrus and posterior cingulate, and right hippocampus. Signal decrease (i.e., negative load effect) was found bilaterally in posterolateral cerebellar regions (left crus II, right crus I), occipital and lingual regions, and superior temporal gyrus, as well as in right precuneus, right IPL, and left middle frontal gyrus. Subsequent analyses of phonological similarity and novelty effects were performed on these load-sensitive ROIs, and are divided into an a priori set and an exploratory set.

Posterior activations in each individual subject (mapped onto their brain) are provided as supplementary material in Online Resource 1.

#### ROI analyses of phonological similarity and sequence novelty: a priori set

To elucidate how activations in regions implicated in the load effect were modulated as a function of novelty and phonological similarity, we conducted ROI analyses for these regions. Note that in these analyses, the load main effect was significant for all regions, and the analyses below were conducted on the high-load trials only. The results of these analyses are summarized in Table 2.

#### Cerebellum

**Fig. 4:**
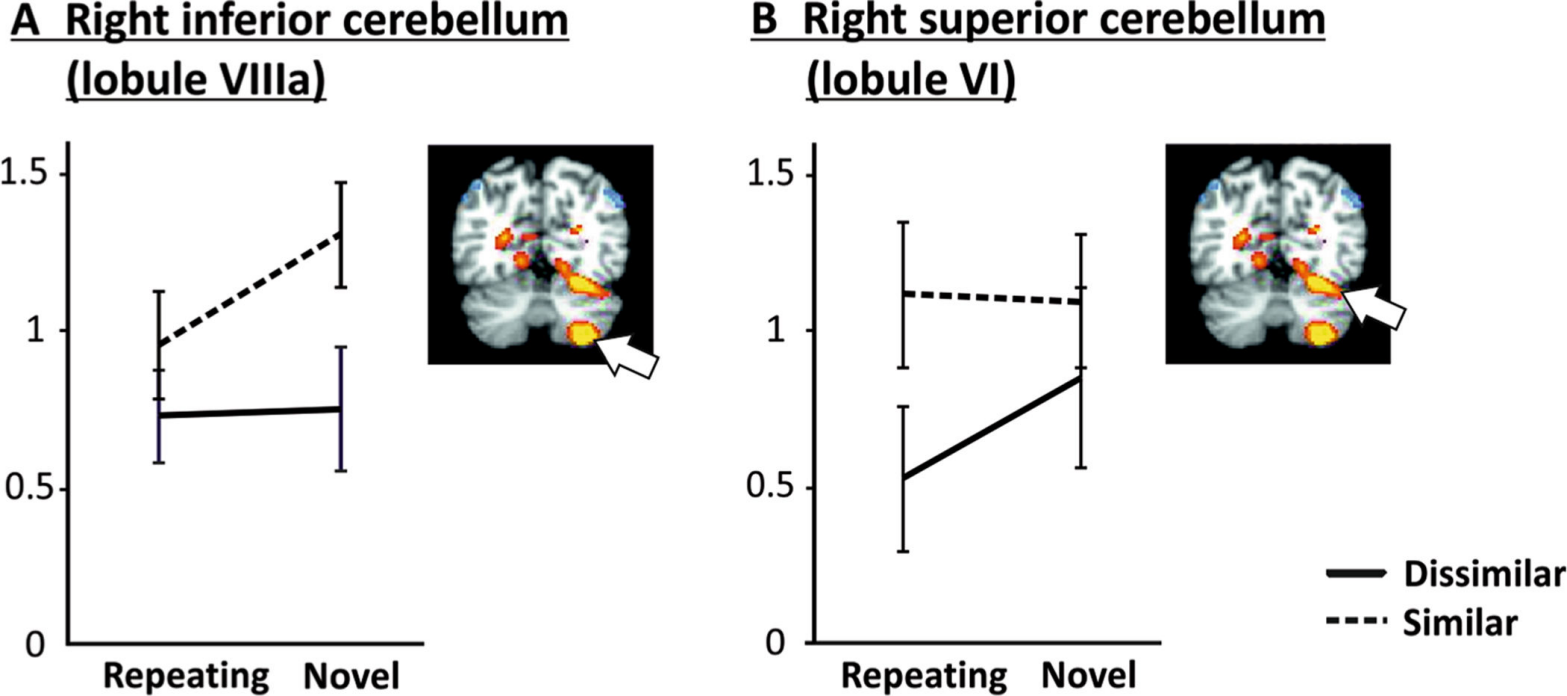
Parameter estimates for cerebellar regions during the maintenance phase of the task according to phonological similarity and novelty in high-load trials. **a** For the right inferior cerebellum, when letter sequences were novel, phonologically similar letters produced significantly greater activation than dissimilar letters. However, the activation for similar letters decreased significantly when repeating sequences were presented. **b** For the right superior cerebellum, activations significantly decreased for phonologically dissimilar letters with repeating sequences compared to phonologically similar letters with repeating sequences

Figure 4 shows average parameter estimates according to phonological similarity and novelty for high-load trials for the right inferior (A) and superior (B) cerebellum. Both these regions showed positive load effects, i.e., increased activation for high relative to low load.

For the right inferior cerebellum (lobule VIIIa), a main effect of similarity (*F*_[1,18]_ = 6.49, *p* = .02) emerged, with increased activation for similar as compared to dissimilar letters. The novelty main effect approached significance (*p* = .07). Planned comparison (1), SNH–SRH, was significant (*t*_18_ = 2.11, *p* = .049), indicating that activation was significantly decreased for repeated sequences of phonologically similar letters. Planned comparison (3), SNH–DNH, was also significant (*t*_18_ = 2.45, *p* = .025), indicating that activation for novel similar letters was significantly greater than for novel dissimilar letters.

The right superior cerebellum exhibited a remarkably different pattern from the inferior cerebellum. Neither similarity nor novelty main effects were observed, although the similarity main effect approached significance (*p* = .07). Only planned comparison (4), SRH–DRH, reached significance (*t*_18_ = 2.16, *p* = .044), indicating that activation in right lobule VI was increased for high-load trials with phonologically similar repeating letters relative to high-load trials with phonologically dissimilar repeating letters.

To further ascertain if the different patterns of activation for the inferior and superior cerebellum noted above were distinctly (and significantly) different from each other, we conducted a repeated-measure ANOVA on the high-load activations with region (inferior, superior), similarity (dissimilar, similar), and novelty (repeated, novel) as within-subject factors. This analysis revealed a significant region × similarity × novelty interaction (*F*_[1,18]_ = 12.27, *p* = .003), verifying that the phonological similarity-dependent effect of sequence repetition described above was significantly different for inferior and superior cerebellar regions.

#### Cerebrum

**Fig. 5:**
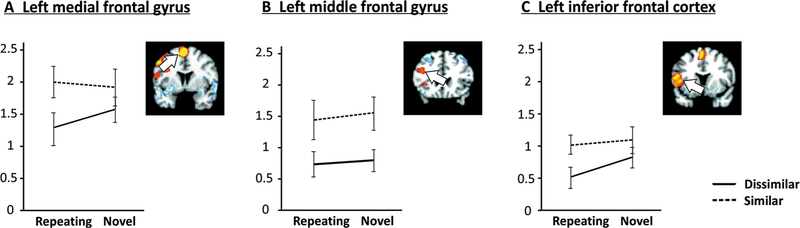
Parameter estimates for cerebral regions during the maintenance phase of the task according to phonological similarity and novelty in high-load trials for regions of interest with positive load effects, i.e., increased activation for high relative to low load

Figure 5 provides average parameter estimates according to similarity and novelty for high-load trials for neocortical regions showing a positive load main effect (see Table 1).

For left medial frontal gyrus (Fig. 5a), analysis yielded a significant main effect of similarity (*F*_[1,18]_ = 9.40, *p* = .007), indicating that activation was increased for similar relative to dissimilar letter sequences. Similar to the superior cerebellum, planned comparison SRH–DRH was significant (*t*_18_ = 2.656, *p* = .016).

A significant main effect of similarity (*F*_[1,18]_ = 20.13, *p* < .001) also emerged for the cluster in left middle frontal gyrus (Fig. 5b). Planned comparisons SNH–DNH and SRH–DRH were both significant (*t*_18_ = 3.684, *p* = .002, and *t*_18_ = 2.76, *p* = .013, respectively) indicating that on high-load trials phonologically similar letters always produced greater activation than dissimilar letters, regardless of whether the sequence was novel or repeating.

All effects and planned comparisons failed to reach significance for the left postcentral gyrus (all *p* > .100). However, a sub-cluster (local maximum in inferior frontal cortex, BA44; Fig. 5c) showed significant main effects of similarity (similar > dissimilar, *F*_[1,18]_ = 14.85, *p* = .001) and novelty (novel > repeating, *F*_[1,18]_ = 6.11, *p* = .024). Planned comparison SRH–DRH was also significant (*t*_18_ = 2.92, *p* = .009), and the DNH–DRH planned comparison approached significance (*t*_18_ = 1.81, *p* = .087).

**Fig. 6:**
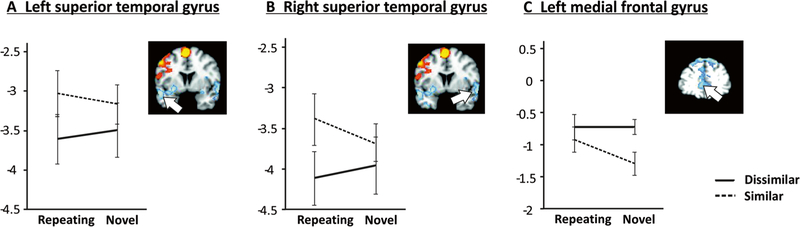
Parameter estimates according to phonological similarity and novelty in high-load trials for regions of interest with negative load effects, i.e., increased activation for low relative to high load

For the left superior temporal gyrus, which exhibited a negative load effect, there was a significant main effect of similarity (*F*_[1,18]_ = 11.35, *p* = .003), reflecting relatively increased activation for similar compared to dissimilar (see Fig. 6a). Planned comparison SRH–DRH was also significant (*t*_18_ = − 2.50, *p* = .022).

#### Exploratory ROI analyses of phonological similarity and sequence novelty: post hoc set

For the remaining 14 clusters exhibiting load-dependent activation, Bonferroni-corrected (according to the number of regions, yielding a significance threshold of *p* < .0036) tests of main effects and planned comparisons were conducted as exploratory analyses. These clusters included 3 regions exhibiting positive load effects in left posterior cingulate, right hippocampus, and right inferior parietal lobule, and 11 regions exhibiting negative load effects in cerebellar left crus II and right crus I, left lingual gyrus, left middle, medial, and superior frontal gyrus, left superior occipital gyrus, right inferior occipital gyrus, right inferior parietal lobule, right precuneus, and right superior temporal gyrus.

One ROI in the post hoc set reached significance at the Bonferroni-corrected threshold: a main effect of phonological similarity was found for the right superior temporal gyrus (*F*_[1,18]_ = 11.73, *p* = .003). Activations approaching significance (i.e., significance at 0.0036 < p < .05) were found in two regions that exhibited a negative load effect: (1) planned comparison SRH–DRH (*t*_18_ = − 3.17, *p* = .005) for the right superior temporal gyrus (Fig. 6b). (2) A main effect of similarity (*F*_[1,18]_ = 6.98, *p* = .017) and planned comparisons SNH–SRH (*t*_18_ = 2.30, *p* = .034) and SNH–DNH (*t*_18_ = 3.09, *p* = .006) for the local maximum in medial frontal gyrus, BA10 (Fig. 6c).

## Discussion

The present study was aimed to further elucidate how the cerebellum contributes to verbal working memory. We reasoned that because the inferior cerebellum has been shown to activate during the maintenance phase of the Sternberg Task (Desmond et al. 1997; Chen and Desmond 2005a, b), where phonological looping occurs, and because increasing the phonological storage demand has been shown to behaviorally impair verbal working memory performance (e.g., Conrad and Hull 1964; Baddeley et al. 1975; Desmond et al. 1997), increased phonological demand ought to be reflected in inferior cerebellar activations. To manipulate phonological store demand, cognitive load (low/high), phonological similarity (similar/dissimilar), and sequence novelty (repeating/novel) were modulated in an fMRI-based Sternberg task. Learning a repeating sequence of study letters was expected to reduce phonological storage demand and, because the cerebellum has been linked in many contexts to learning and plasticity (e.g., Molinari et al. 2008; Ramnani et al. 2000), also reduce phonological storage-related right inferior cerebellar activations. This effect was hypothesized to be modulated by phonological similarity of the study letters, with overall increased right inferior cerebellar activations for similar relative to dissimilar study letters, and greater repetition-related decreases in activation for similar relative to dissimilar letters, especially at higher memory load. Our results not only confirmed these hypotheses but also showed that the load-dependent inferior cerebellar activation was among the highest magnitude activations for this task (Table 1).

Behavioral data (RTs and accuracy) replicated well-established load effects in working memory, with higher RTs and decreased accuracy for trials with high as compared to low cognitive load (e.g., Peterburs et al. 2016; Marvel and Desmond 2012; Kirschen et al. 2010). Notably, for accuracy, this effect emerged as a function of phonological similarity, with higher accuracy for dissimilar than for similar high-load trials, while there was no difference for low-load trials, which was likely due to a ceiling effect. This finding is in line with earlier reports of decreased verbal working memory performance for similar sounding phonemes (Baddeley 1965; Conrad 1964) and phonologically similar compared to dissimilar consonants in a two-back task (Sweet et al. 2008) and can be attributed to phonemic interference, leading to increased phonological storage demand for phonologically similar content. Somewhat contrary to our expectations, there was no novelty main effect of accuracy, although the near-significant load by novelty interaction did suggest that repeating sequences affected accuracy in high- but not low-load trials, possibly again reflecting a ceiling effect in the low-load condition. Analysis of median RT showed a linear decrease in RT across the three runs for trials with a repeating sequence irrespective of phonological similarity. Since such a decrease in RT was not found for trials with novel sequences, this confirms that the repeating sequences were implicitly learned over the course of the task. This result is in line with previous reports of implicit learning of perceptual sequences in the absence of motor sequencing (e.g., Dennis et al. 2006). Interestingly, novelty effects also emerged as a function of similarity and load. Repeating sequences significantly decreased RTs for high-load trials, in particular for the similar condition. This result pattern suggests that, as predicted, implicit learning of repeating sequences reduced demand on the phonological store and related processes of articulatory monitoring and error correction during rehearsal more substantially for similar as compared to dissimilar study letters. Before discussing the present imaging results, it is worth noting that these RT differences were unlikely to affect brain activation patterns, as the maintenance phase preceded probe presentation, and probe onset was not predictable.

With regard to functional data, we replicated the typical load effect in verbal working memory, with increased activation for high relative to low load in several cerebellar and neocortical regions: right superior and inferior cerebellum (lobules VI and VIIIa), IPL, and left inferior frontal cortex (e.g., Chen and Desmond 2005a, b; Kirschen et al. 2005; Peterburs et al. 2016). Negative load effects, i.e., decreased activation for high vs. low load, were found for IPL, precuneus, and middle frontal cortical regions. Interestingly, these regions have been associated with the default mode network (DMN), a large-scale brain network shown to reduce activation in periods of focused attention, e.g., during performance of a particular task, and to increase activation in periods of wakeful rest and relaxed attention (e.g., Buckner et al. 2008 for an overview). In line with this, previous work has reported decreased DMN activation in working memory tasks (e.g., Koshino et al. 2014). Greater deactivation for similar compared to dissimilar letters in these regions that was observed in the present study corresponds to earlier findings (Sweet et al. 2008) and has been interpreted in terms of greater focusing of attention to maintain performance levels in more difficult task conditions.

Superior temporal regions, i.e., primary sensory regions for auditory processing, were also relatively deactivated for high compared to low cognitive load. It is important to point out that auditory input was limited to the encoding phase in the present variant of the Sternberg task. Suppression of primary sensory regions during the maintenance phase has previously been reported and hypothesized to enable protection of short-term memory representations from being overwritten by inhibiting the encoding of interfering sounds (Linke et al. 2011). However, the present findings of bilateral superior temporal deactivation as a function of phonological similarity and novelty are only partly compatible with this notion. With more interference for phonologically similar items, deactivation should have been stronger for similar repeating high load compared to dissimilar repeating high load, but the opposite pattern was observed (Fig. 6a, b). Of note, this pattern was different from all other negative load regions reported in Table 1, which exhibited either comparable levels of activation for similar and dissimilar letters or more deactivations for similar relative to dissimilar letters (Fig. 6c). More research is needed to further elucidate processing in superior temporal regions as a function of phonological similarity and novelty.

Crucially, the present findings support and further elucidate the notion of differential roles of the superior and inferior cerebellum in verbal working memory. Previous work has reported pronounced lobule VI activations, in concert with activations in posterior frontal regions, especially in the encoding phase of the Sternberg task (e.g., Chen and Desmond 2005a, b; Chein and Fiez 2001; Peterburs et al. 2016), attributing these to processes of articulatory control. In contrast, the inferior cerebellum was most engaged in the maintenance phase and has been linked to phonological storage (e.g., Desmond et al. 1997; Chen and Desmond 2005a). In the present study, we observed pronounced activations in both regions during maintenance, likely because our task— which involved auditory stimulus presentation during encoding—did not allow for visual strategies for encoding and thus posed higher articulatory demand.

In accordance with involvement in articulatory control, the superior cerebellum (lobule VI) was sensitive to phonological similarity as a function of load and novelty. The observed activation patterns in lobule VI (Fig. 4b) were similar to left posterior frontal regions (BA 6 and BA 44, Fig. 5a, c, respectively) which have been shown to be functionally coupled with the superior cerebellum especially during encoding (e.g., Chen and Desmond 2005a, b; Chein and Fiez 2001). Interestingly, and somewhat contrary to our expectations, implicit acquisition of repeating sequences appears to have decreased activations for dissimilar, but not similar high-load trials, leading to the significant SRH–DRH and non-significant SNH–DNH contrast pattern apparent in Table 2. One possible explanation for this pattern is that for frontal and superior cerebellar regions, which have been associated with articulatory task requirements, there may be greater variability in the articulatory trajectory for phonologically dissimilar letters than for similar letters, thereby allowing for a greater amount of plasticity that is achievable for the repeating articulatory sequence.

In line with our predictions, the present results yielded increased activation in the inferior cerebellum (lobule VIIIa) for similar relative to dissimilar letters, reflecting greater phonological storage demand for similar sounding letters or phonemes. This extends findings from a previous study on phonological similarity effects (Sweet et al. 2008), which reported increased cerebellar activations only in superior regions for a phonologically similar compared to a dissimilar n-back task. Importantly, in contrast to the present work, the imaging protocol in this study may not have been optimized for the cerebellum, raising the possibility that inferior portions of the cerebellum were not adequately captured.

Interestingly, lobule VIIIa also presented with a unique activation pattern with regard to novelty (see Fig. 4b). Activation was decreased for repeated vs. novel for phonologically similar high-load trials, yielding significant SNH–SRH as well as SNH–DNH contrasts. This is consistent with implicit learning of the repeated letter sequence and suggests that in verbal working memory, lobule VIIIa may generate association-based predictions of letter sequences that would reduce the likelihood of phonological loop failure before the retrieval phase of the Sternberg task. Along these lines, our results support sequence detection accounts of cerebellar function (Molinari et al. 2008). The present results are also in line with the notion that cerebellar dysfunction and resulting deficits in sequence acquisition and reproduction may be critical in patients with dyslexia (Nicolson et al. 2001). In a positron emission tomography (PET) study, Nicolson et al. (1999) investigated cerebellar activation during reproduction of a pre-learned finger tapping sequence and during acquisition of a novel tapping sequence (versus a rest condition) in dyslexic adults and healthy control subjects. Results showed reduced (particularly right) cerebellar activations in dyslexia patients relative to controls both during performance of a pre-learned and learning of a novel sequence. Moreover, while controls showed relatively increased cerebellar activation for performance of the pre-learned sequences vs. rest and for learning of a novel sequence vs. rest, this pattern was absent (pre-learned sequences) or substantially reduced (novel sequences) in the dyslexia group. Interestingly, activation in frontal regions was increased in the dyslexic relative to the control group, possibly indicating functional compensation. Generally, these findings provide direct evidence that abnormal cerebellar sequencing functions may link to deficits in phonological and articulatory processing.

In the present study, the only other brain region aside from lobule VIIIa in which the SNH–SRH and SNH–DNH contrasts were prominent was a left middle frontal cluster in BA10 that showed a negative load effect and reduced deactivation for similar repeating high-load trials (see Fig. 6c). Although this region was identified in exploratory analyses, and statistical tests did not survive Bonferroni correction, a meta-analysis of n-back studies identified BA10 as one of the brain regions consistently activated across all included studies (Owen et al. 2005). In a comprehensive review article, Ramnani and Owen (2004) posited that the frontopolar cortex is recruited for tasks that involve several discrete cognitive processes, e.g., integration of the results of two or more separate cognitive operations to achieve a higher order goal. It stands to reason that the present variant of the Sternberg task involved integration of different cognitive processes, e.g., sequence learning (albeit subconscious, as none of the subjects reported having become aware of the sequence), articulatory and interference control, attention allocation, and phonological storage. It is thus reasonable to speculate that BA10 might be part of a network supporting phonological storage operations, in line with arguments posed by Buchsbaum and D’Esposito (2008), and that activation in BA10 might reflect integration of sequence-related input from the inferior cerebellum. However, another logical target for the phonological and repetition information of the inferior cerebellar output would be the superior temporal gyrus, a structure involved in the encoding of the auditory stimuli and whose maintenance activation was distinctly different for repeated similar vs. dissimilar letters.

In summary, the present study found the typical effects of cognitive load and phonological similarity in several cerebellar and neocortical brain regions as well as in behavioral data (accuracy and response time). Importantly, activations in superior and inferior cerebellar regions were differentially modulated as a function of similarity and sequence novelty, indicating that particularly lobule VIIIa may contribute to verbal working memory by sequence learning/detection, allowing it to generate predictions of letter sequences and thereby reducing the likelihood of phonological loop failure before retrieval. The present study thus supports sequencing accounts of cerebellar function as a mechanism for providing predictions that benefit neocortical regions for motor or non-motor functions.

## Supplementary Material

Supp

## Figures and Tables

**Table 1:** MNI coordinates of activation maxima for the load contrast (high > low)

Brain region	X	Y	Z	SPM {Z}	Size (mm^3^)
Significant activations for high > low load during maintenance					
Cerebellum					
Right inferior cerebellum (lobule VIIIa)	24	− 68	− 58	5.73	586
Right superior cerebellum (lobule VI)	24	− 66	− 18	4.76	597
Cerebrum					
Left medial frontal gyrus (BA6)	− 2	4	64	5.31	787
Left middle frontal gyrus (BA9)	− 44	24	28	3.67	172
Left postcentral gyrus (BA3)^a^	− 52	− 6	46	4.39	1623
Left posterior cingulate (BA30)	− 24	− 60	12	4.39	571
Right hippocampus	36	− 36	− 6	4.14	180
Right inferior parietal lobule (BA40)	42	− 34	50	3.81	142
Significant deactivations for high > low load during maintenance					
Cerebellum					
Left crus II	− 20	− 80	− 40	4.39	613
Right crus I	28	− 76	− 32	3.9	257
Cerebrum					
Left lingual gyrus (BA18)	− 22	− 94	− 6	5.09	422
Left middle frontal gyrus (BA6)^b^	− 30	24	56	5.56	4692
Left superior occipital gyrus (BA19)	− 38	− 78	34	4.3	156
Left superior temporal gyrus (BA22)	− 48	− 18	4	5.69	1960
Right inferior occipital gyrus (BA17)	26	− 98	− 4	4.26	144
Right inferior parietal lobule (BA40)	48	− 60	48	3.73	248
Right precuneus (BA31)	10	− 44	38	4.64	559
Right superior temporal gyrus (BA22)	56	− 14	2	5.75	3389

a Local maxima in BA44 in inferior frontal gyrus (− 52, 10, 16) and precentral gyrus (− 44, 10, 6)

b Local maxima in medial frontal gyrus BA10 (− 10, 56, 2) and superior frontal gyrus BA8 (− 8, 46, 50); *BA* Brodmann area

**Table 2:** Summary of ANOVA results for a priori regions of interest

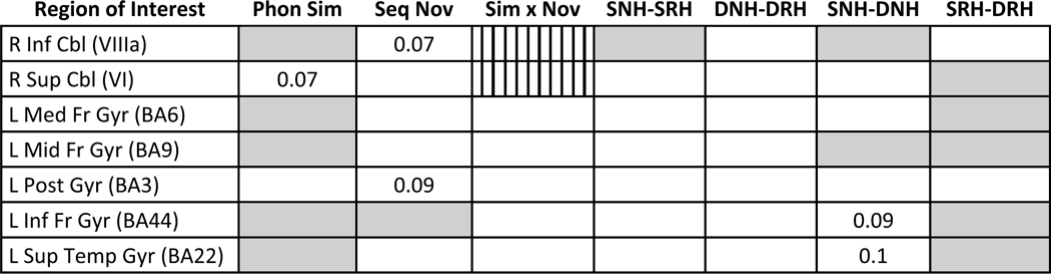

Gray-shaded cells indicate statistically significant effects (*p* < .05) for main effects of phonological similarity (Phon Sim), sequence novelty (Seq Nov), the phonological similarity × sequence novelty interaction (Sim × Nov), and four planned contrasts, where *S* phonologically similar, *D* dissimilar, *N* novel sequences, *R* repeated sequences, and *H* high memory load. Note that the Sim × Nov interaction differed for superior and inferior cerebellum (vertically striped cells), as indicated by the significant region × similarity × novelty interaction

*ROI* region of interest, *L* left, *R* right, *Cbl* cerebellum, *Inf* inferior, *Sup* superior, *Med* medial, *Mid* middle, *Fr* frontal, *Post* postcentral, *Temp* temporal, *Gyr* gyrus, *BA* Brodmann area
