# Supplementary material for: Cerebellar contributions to sequence prediction in verbal working memory
Source: Brain Struct Funct. Author manuscript; Available in PMC 2019 Feb 26. (PMC6373538; doi:10.1007/s00429-018-1784-0)

SUPPLEMENTARY MATERIAL

Cerebellar contributions to sequence prediction

in verbal working memory

Jutta Peterburs[1,2], Laura C. Blevins[1,3], Yi-Shin Sheu[1], John E. Desmond[1]

[1]Department of Neurology, Division of Cognitive Neuroscience, Johns Hopkins University School of Medicine, Baltimore, Maryland, USA
[2]Department of Biological Psychology, Institute of Experimental Psychology, Heinrich-Heine-University Duesseldorf, Germany
[3]Department of Psychology, American University Washington, DC, USA

Corresponding author: Jutta.Peterburs@hhu.de

ONLINE RESOURCE 1: Individual-level load effect

In order to ascertain that the activation found in the superior cerebellum in the group-level analysis was not due to "bleed-over" from inferior temporal lobe sources due to the proximity of these structures, or an artifact of the averaging process, we generated images of the posterior activations in each individual subject (S1-S19), mapping the activations onto their individual brains. These images (see below) provided convincing evidence that the superior cerebellar activation is not an artifact of the averaging process, but rather is derived from activation originating in the superior cerebellum on a subject by subject basis.

Please note that for illustration purposes, the images are thresholded at $p<.001$ for most subjects (and at $p<.01$ or $p<.05$ for a few, depending on the strength of the effect). In general, there was rather good consistency between subjects and high overlap with load-modulated cerebellar regions identified in previous studies (e.g., Awh et al., 1996; Desmond et al., 1997; Chen & Desmond, 2005; Durisko & Fiez, 2010; Jonides et al., 1997; Fiez et al., 1996; Grasby et al., 1994; Paulesu et al., 1993, 1995).

S01
p<.001

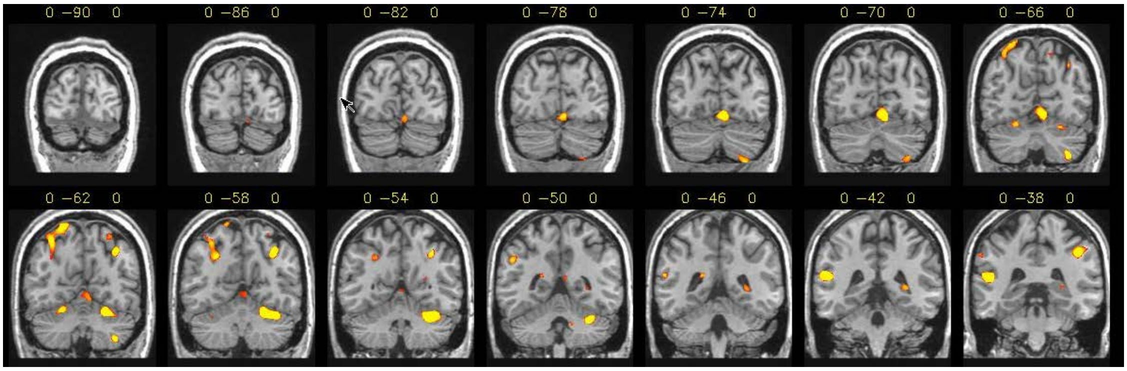

S02
p<.001

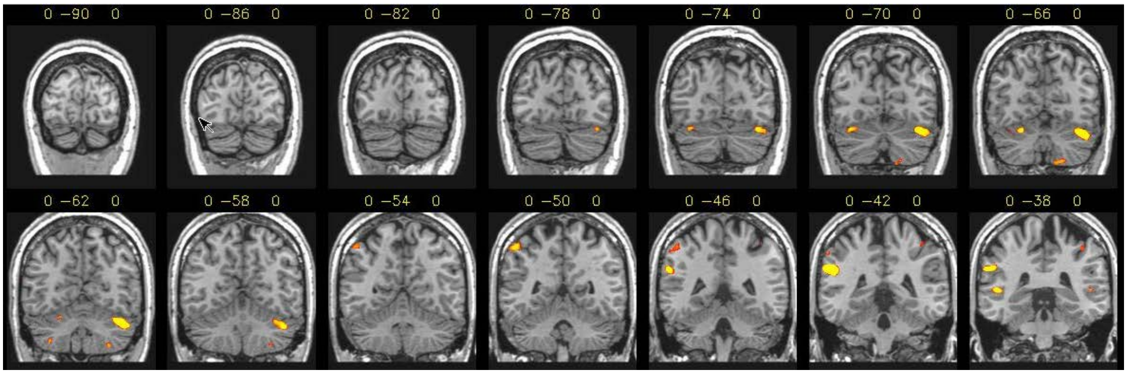

S03
p<.001

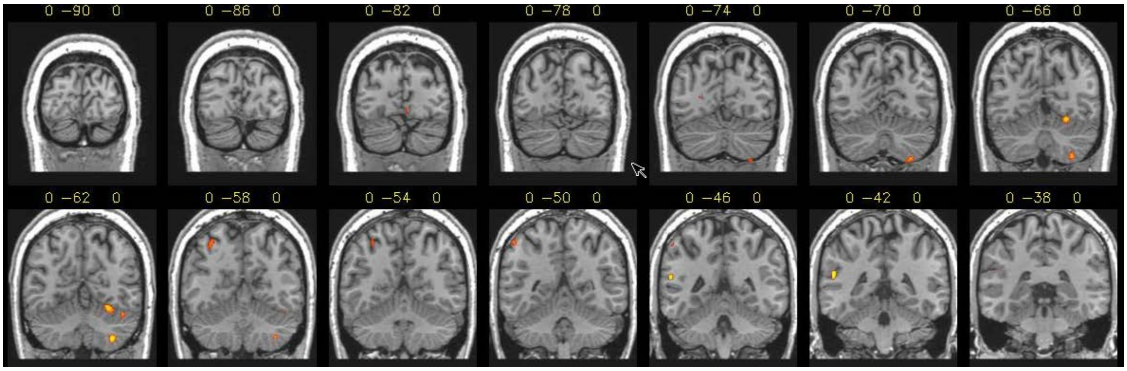

S04
p<.001

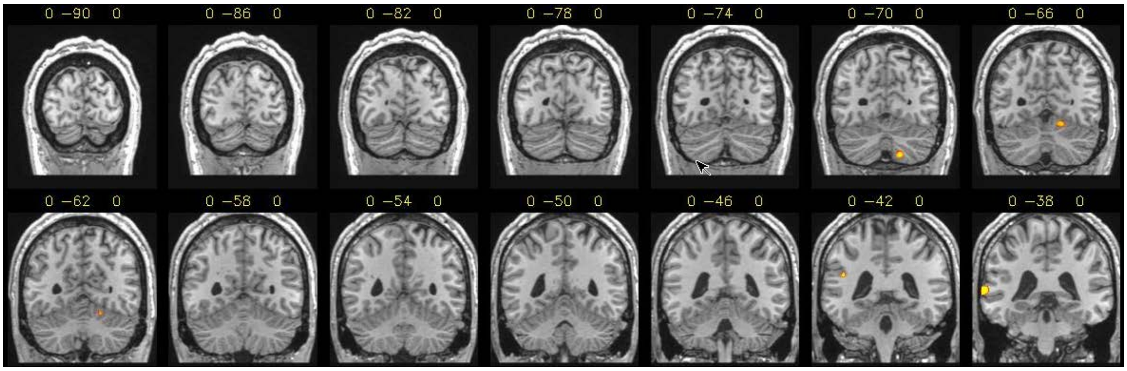

S05
$p<.05$

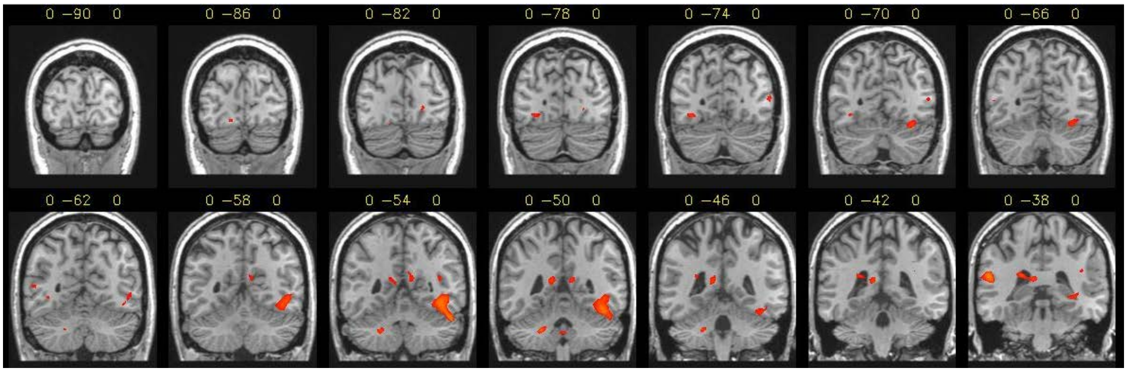

S06
$p<.01$

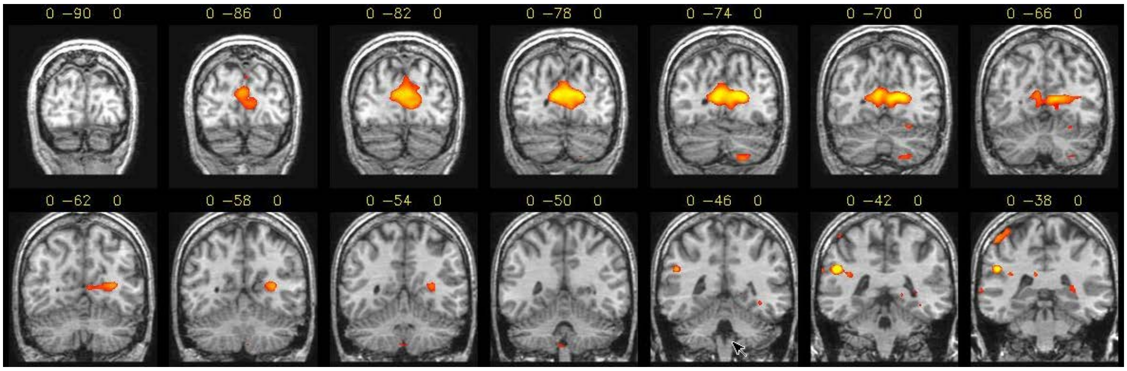

S07
$p<.01$

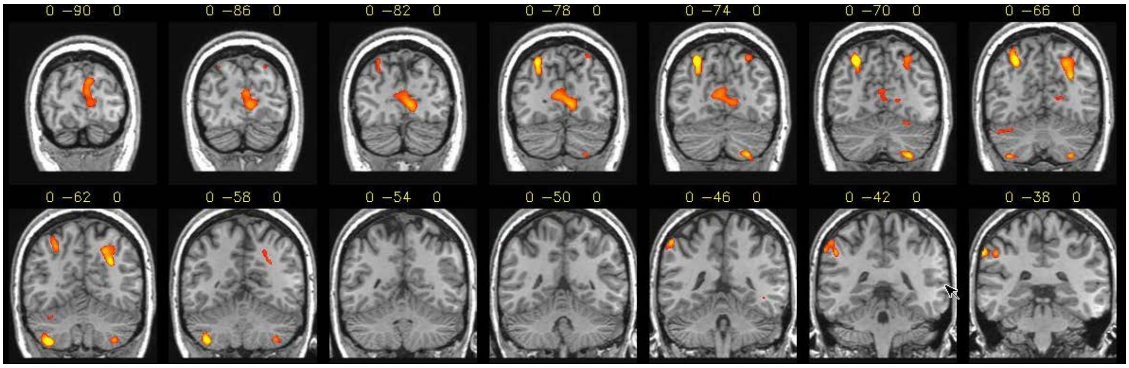

S08
$p<.001$

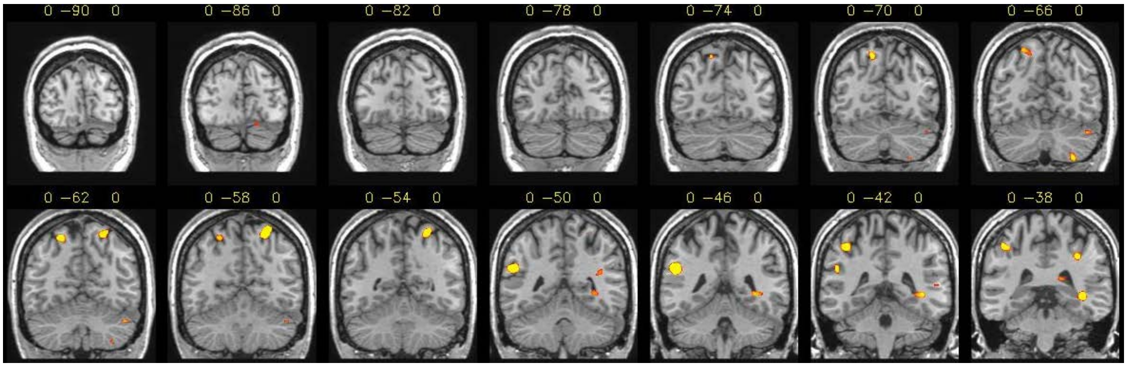

S09
p<.001

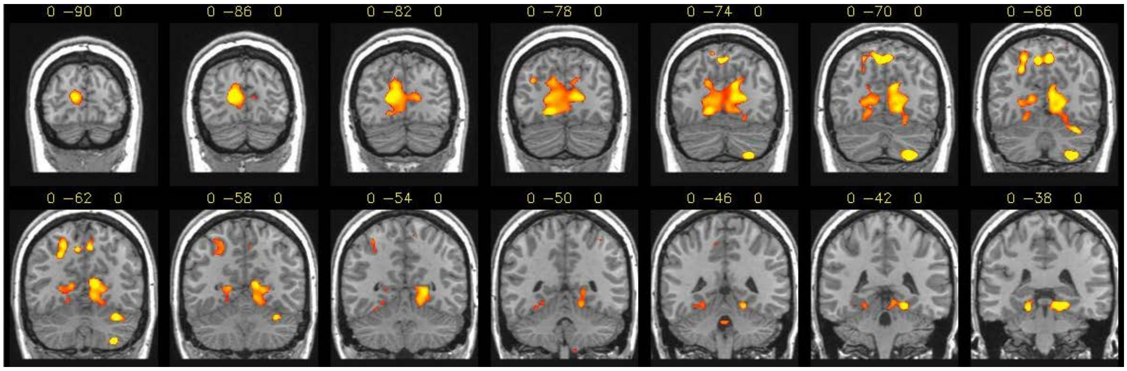

S10
p<.05

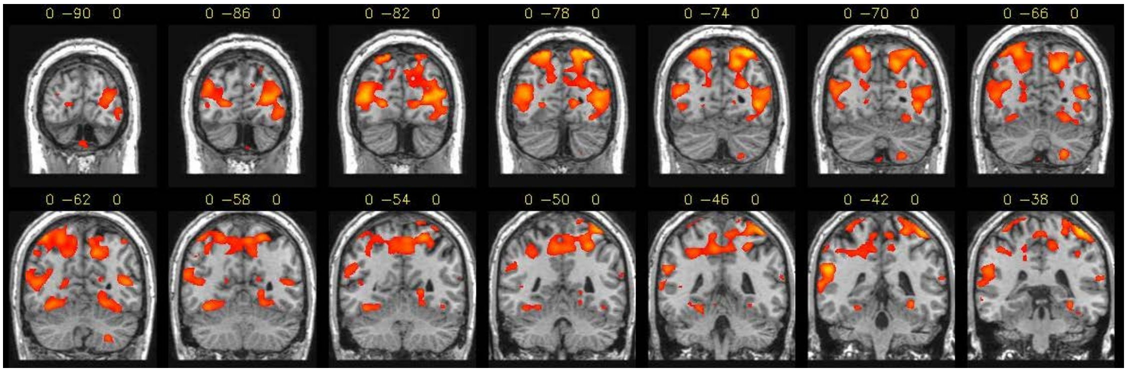

S11
p<.001

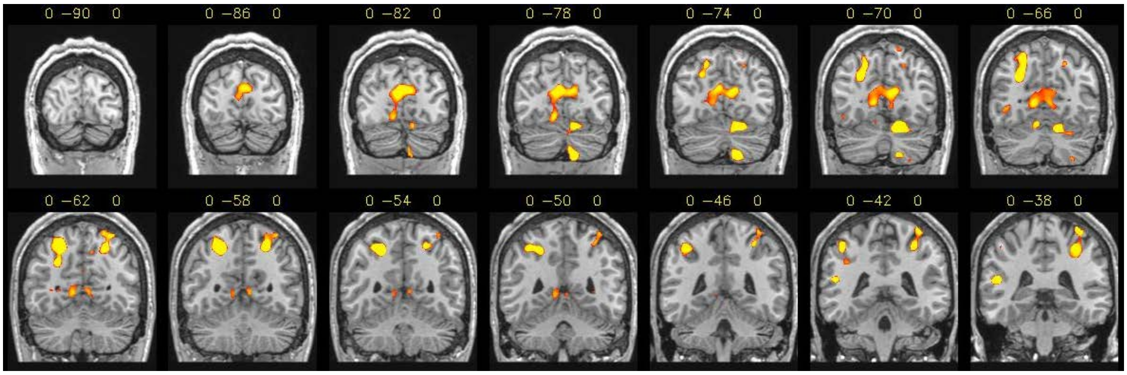

S12
p<.001

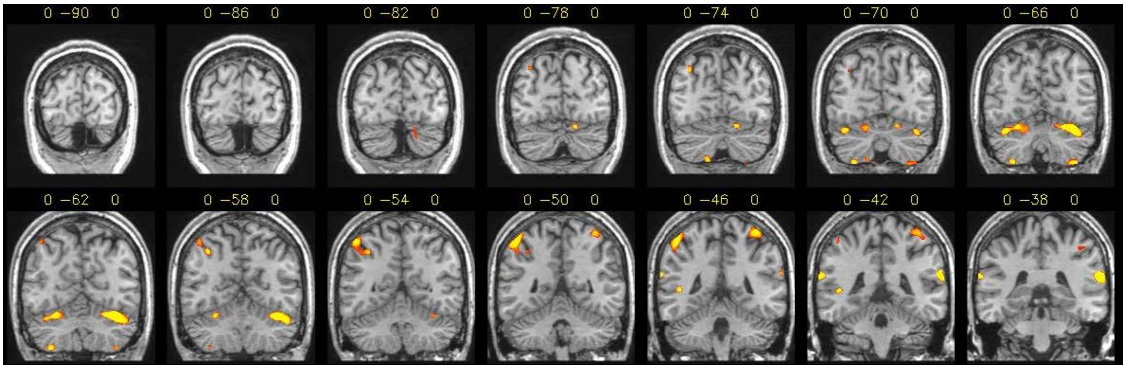

S13
p<.05

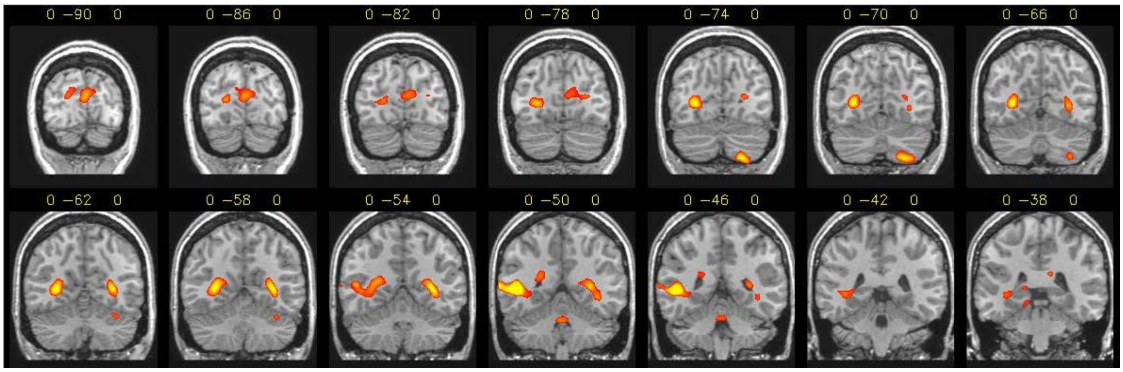

S14
p<.001

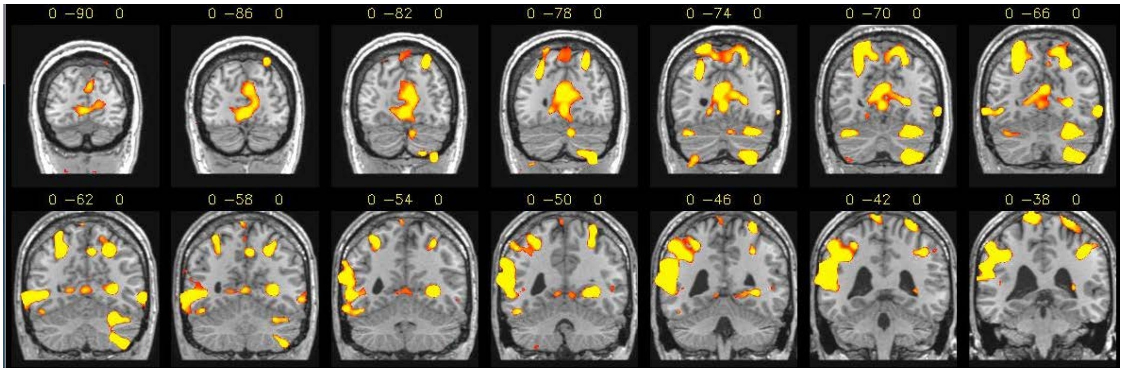

S15
p<.001

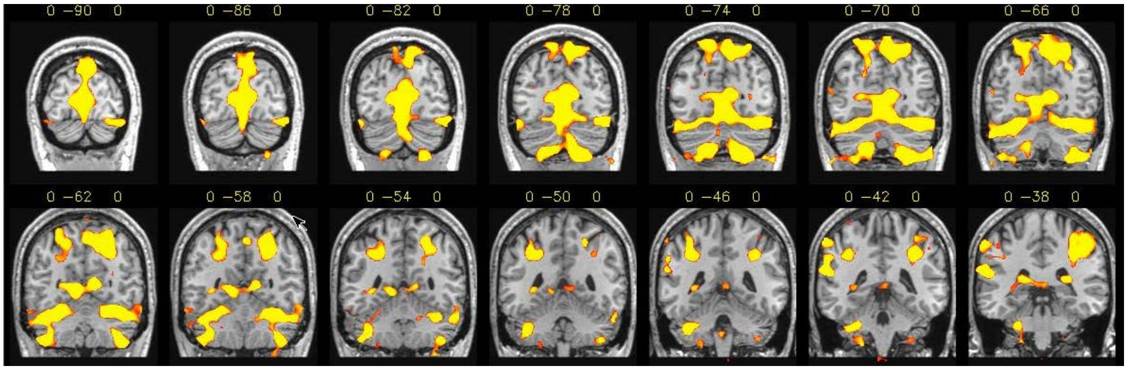

S16
p<.001

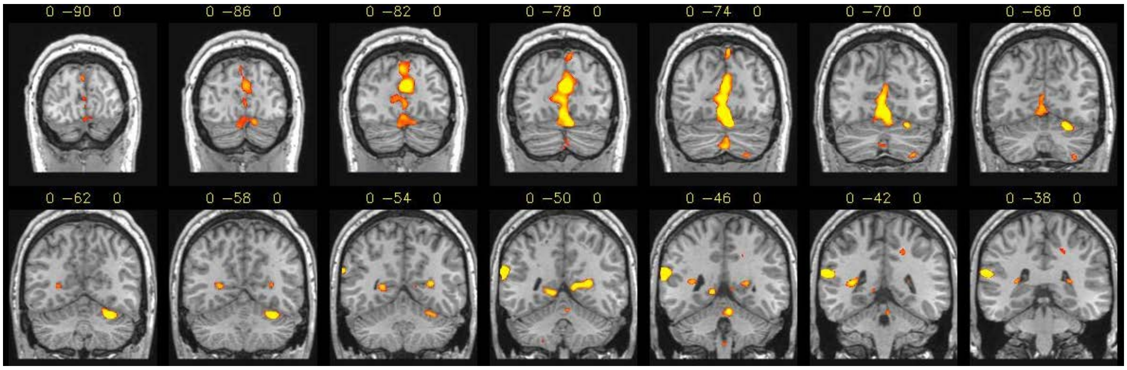

S17
p<.05

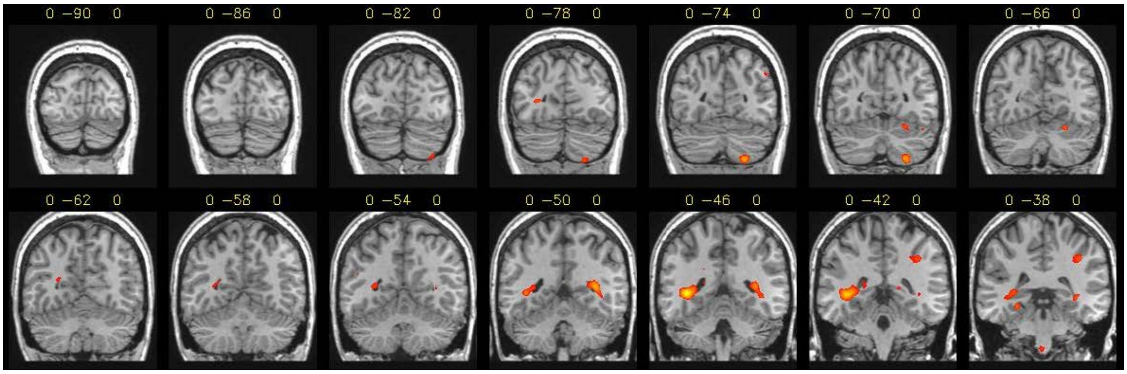

S18
p<.001

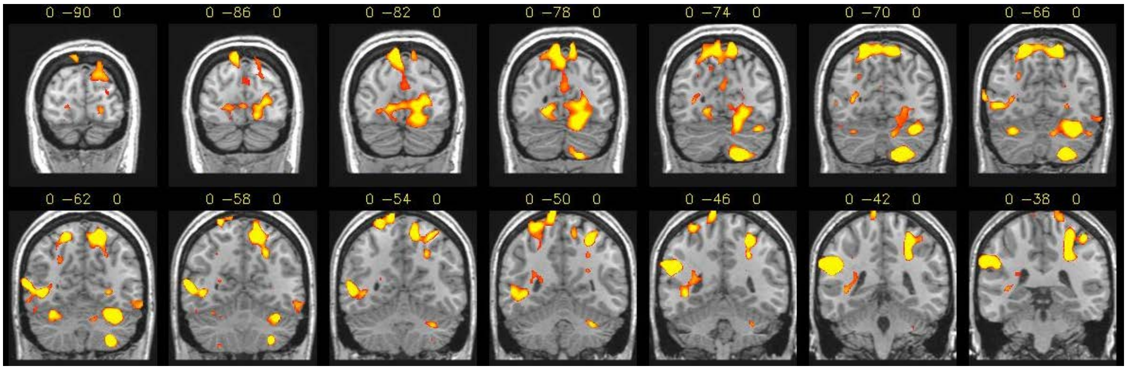

S19
p<.001

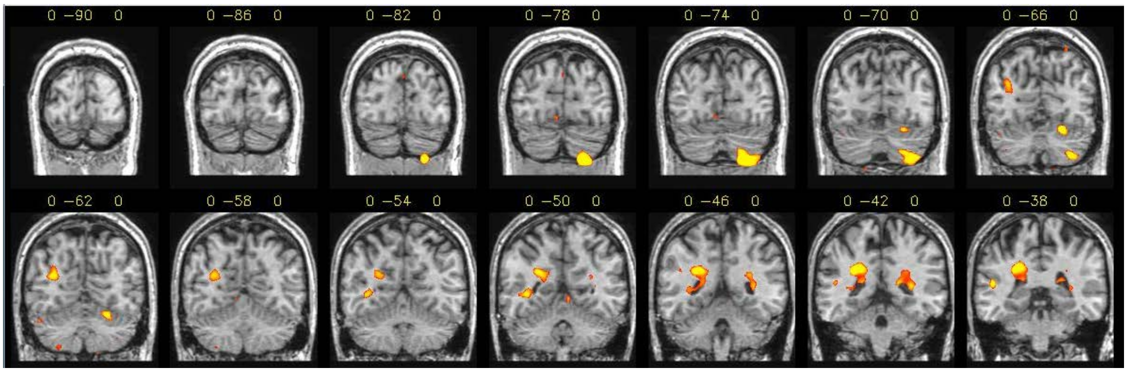

Supplement: Supp [file NIHMS1012638-supplement-Supp.pdf]
